# *Ginkgo biloba *extract (EGb761) inhibits mitochondria-dependent caspase pathway and prevents apoptosis in hypoxia-reoxygenated cardiomyocytes

**DOI:** 10.1186/1749-8546-6-8

**Published:** 2011-02-23

**Authors:** Jiangang Shen, Waisin Lee, Yong Gu, Yao Tong, Peter CW Fung, Li Tong

**Affiliations:** 1 School of Chinese Medicine, The University of Hong Kong, 10 Sassoon Road, Pokfulam, Hong Kong SAR, China; 2 Department of Medicine, The University of Hong Kong, 21 Sassoon Road Pokfulam, Hong Kong SAR, China; 3 School of Chinese Medicine, Southern Medical University, Guangzhou 510515, China

## Abstract

**Background:**

EGb761 is a standard extract from the leaves of *Ginkgo biloba *(*Yinxing*) containing ginkgo-flavone glycosides and terpenoid. The flavonoid components of EGb761 scavenge free radicals and protect myocardia from ischemia-reperfusion injury. The present study aims to determine the effects of the active compounds of EGb761 on mitochondria-dependent caspase pathway.

**Methods:**

Cardiomyocytes were exposed to 24 hours of hypoxia and four hours of reoxygenation, and pretreated with EGb761, bilobalide and quertcetin. By using immunoblot, immunofluorescent, biochemical and flow cytometry techniques, we compared the effects of EGb761 and its representative constituents including quercetin and bilobalides on regulating mitochondria-dependent caspases signal pathway and apoptotic cell death in the hypoxia-reoxygenated cardiomyocytes.

**Results:**

Pretreatment with EGb761 significantly inhibited the release of cytochrome c from mitochondria, the expression of caspase-3, cleavage activities of caspases and attenuated apoptotic cell death. The effects of quercetin on the release of cytochrome c, the cleavage activities of caspases and cell death were similar to those of EGb761 but better than those of bilobalide.

**Conclusion:**

The antioxidant constituents of EGb761 such as quercetin contribute to the cardioprotective effects of EGb761 and inhibit the mitochondria-dependent caspase pathway. It is possible that the mitochondria-dependent caspase pathway may be one of the molecular targets of EGb761 against myocardial ischemia-reperfusion injury.

## Background

EGb761 is a standard extract from the leaves of *Ginkgo biloba *(*Yinxing*) containing 24% ginkgo-flavone glycosides (*eg *kaempferol, quercetin and isorhamnetin derivatives) and 6% terpenoid (*eg *ginkgolides A, B, C, J and bilobalide) [1]. The cardioprotective effects of EGb761 have been demonstrated in various *in vivo *and *in vitro *animal models and humans. The flavonoid components of EGb761 scavenge superoxide, hydroxyl radicals and nitric oxide (NO) and protect myocardia from ischemia-reperfusion injury [2-5]. The terpenoid constituents of EGb761 also showed their cardioprotective effects independent from the free radical-scavenging properties [6]. Therefore, it is necessary to further elucidate whether the cardioprotective mechanisms of EGb761 are attributed to its flavonoids or terpenoid conponents in the prevention of myocardial ischemia-reperfusion injury.

Mitochondria-dependent caspase-3 pathway is one of the critical signal pathways in apoptotic cell death during myocardial ischemia-reperfusion injury [7]. The mitochondrial apoptotic pathway plays a pivotal role in the apoptotic cell death [8]. The release of cytochrome c from mitochrondria in response to proapoptotic signals has been suggested as an initiating event in the apoptotic process [9]. Cytochrome c released from mitochondria is associated with apoptosis protease activating factor (Apaf-1) and pro-caspase-9, triggering the activation of caspase-3 and resulting in cell death [9]. Reactive oxygen species (ROS) generated from the ischemia-reperfused cardiomyocytes trigger apoptotic cell death [10]. ROS can lead to the release of cytochrome c and precursors of caspases from the mitochondria into the cytoplasm [11]. Ischemia-reperfusion initiates the release of cytochrome c within minutes and the program of apoptotic cell death within hours in the cardiomyocytes [12]. Therefore, antioxidant therapy targeting the mitochondrial apoptotic pathway may be an important strategy in the treatment of myocardial ischemia-reperfusion injury. Recent studies show that EGb761can effectively and extensively counteract the cardial toxicity of doxorubicin *via *preventing the activation of the p53-mediated, mitochondrion-dependent apoptotic signaling pathway [13,14]. EGb761 also protects against mitochondrial dysfunction in platelets and hippocampi in ovariectomized rats [15]. However, it is unclear yet whether EGb761 can regulate mitochondria-dependent caspases pathway in ischemia-reperfused cardiomyocytes.

In this study, we selected two representative constituents of EGb761, namely quercetin and bilobalide, and compared their effects on the release of cytochrome c from mitochondria, the expression of caspase 3, the cleavage activities of caspases and apoptotic cell death.

## Methods

### Cell culture and drug treatment

Neonatal Wistar rat cardiac myocytes were isolated and cultured according to the method described in our previous report [16]. The rats were obtained from the Laboratory Animal Unit of the University of Hong Kong. Animal housing, care and application of experimental procedures were all in accordance with the institutional guidelines and approved by the University Committee on the Use of Live Animals in Teaching and Research for the University of Hong Kong. Briefly, after anesthesia, the hearts from Wistar rats (aged 2-3-days) were minced and dissociated with 0.06% trypsin (Sigma, USA). The dispersed cells were incubated on 100 mm culture dishes for 15 minutes at 37°C with 100% relative humidity in a CO_2 _incubator. Non-attached viable cells were collected and incubated in Dulbecco's modified Eagle's medium (DMEM, Invitrogen, USA) supplemented with 10% fetal calf serum (FCS), penicillin (50 U/ml) and streptomycin (50 μg/ml) for six hours, followed by incubation in the same media supplemented with 10^-6 ^mol/L cytosine arabinoside (Ara C, Sigma, USA) for 48 hours to reduce the rate of non-myocytes. The purity of the isolated cardiomyocytes was identified to be more than 90%. The isolated cardiomyocytes were cultured in high glucose DMEM supplemented with 10% heat-inactivated FCS, 1% glutamine, 50 U/ml penicillin and 50 μg/ml streptomycin and incubated at 37°C with 100% relative humidity in a CO_2 _incubator for three days. To mimic ischemia, we placed the medium with low glucose DMEM supplemented with 1% FCS, 1% glutamine, 50 U/ml penicillin and 50 μg/ml streptomycin. The cardiomyocytes were incubated at 37°C in an air-tight incubator from which normal air was removed by a vacuum pump (GAST DOA-P184-BN, USA) and replaced by 1% O_2_/5% CO_2 _balanced by 95% N_2_. The cardiomyocytes were cultured under hypoxia for twenty-four hours. To mimic reperfusion, we placed the medium with the high glucose medium supplemented with 10% FCS again and replaced the gas with 95% air/5% CO_2 _for four hours. Corresponding control cells were incubated under the same conditions but perfused with air (ambient). Prior to hypoxic treatment, EGb761 (1, 10, 100 μg/ml, IPSEN Institute, France) and its components quercetin and bilobalide (1, 10, 100 μg/ml, Sigma, USA) were added respectively into the media. To confirm whether the antioxidant therapy reduced the release of cytochrome c from the cardiomyocytes, prior to hypoxia treatment we added MnTMPyP (5 μM; A G Scientific, USA), a superoxide dismutase (SOD) mimic, to the culture medium to dismutate cellular superoxide.

### Electrophoretic analysis of DNA fragmentation

After hypoxia and reoxygenation treatment, the cells were washed twice with phosphate buffered saline (PBS), precipitated by centrifugation and incubated with lysis buffer [0.2M Tris-HC1, pH8.0; 0.1 M sodium ethylenediaminetetracetic (Na_2_EDTA); 1% SDS and 100 mg/L proteinase K] for four hours at 55°C. The nuclear lysates were extracted twice with phenol and then extracted with an equal volume of phenol-chloroform-isoamyl alcohol (25:24:1). DNA was precipitated with 5 mol/L sodium chloride and ethanol overnight at -20°C and spun down at 6,000 *g *with Beckman Coulter Avanti-JE Centrifuge (USA) for 10 minutes at 4°C. The DNA was incubated with Tris-DETA buffer (10 mM Tris-HC1, pH7.5; 1 mM Na_2_EDTA) containing 20 mg/L RNAse A for one hour at 37°C and finally extracted twice with an equal volume of phenol-chloroform-isoamyl alcohol (25:24:1). After washed with 75% ethanol, DNA samples were analyzed with electrophoresis on 1.5% agarose gels with TAE buffer (40 mM Tris-HC1, 20 mM acetic acid and 1 mM Na_2_EDTA). The gel was stained with 0.5 μg/ml ethidium bromide and photographed with UV transillumination.

### Detection of DNA fragmentation by ELISA

Cellular DNA fragmentation was determined with the cell death detection ELISA reagents (Boehringer Mannheim, Germany) according to the manufacturer's instructions. The DNA fragmentation was expressed with the enrichment of histone-associated mono- and oligonucleosomes released into the cytoplasm. The enrichment factor (EF) was calculated with absorbance at 405 nm.

### Caspase cleavage assay

Caspase activity was measured as previously described [17]. Briefly, cells were washed with PBS and suspended in 500 μl of lysis buffer containing 50 mM Tris-HCl pH7.4; 1 mM Na_2_EDTA; 10 mM sodium ethylene glycol tetraacetic (EGTA) and 10 mM digitonin at 4°C for 30 minutes. Lysates were centrifuged at 7200 g with Beckman Coulter Centrifuge (USA) for 10 minutes, and the supernatant containing 50 μg of protein was incubated with 50 μM Ac-DEVD-AMC (Calbiochem, USA) at 37°C for 30 minutes. The release of 7-amino-4-methylcoumarin (AMC) was measured in a fluorescence spectrophotometer (Hitachi F-3010, Japan; excitation 380 nm; emission 460 nm). The enzyme activity was expressed as fluorescent units per minute per milligram of protein. The protein concentration of the supernatant was determined by the Bradford method.

### Western blot analysis

Cytochrome c release from mitochondria into the cytosol was measured with Western blot analysis. Cells were harvested in buffer A containing 20 mM HEPES (pH7.5), 10 mM KCl, 1.5 mM MgCl2, 1.0 mM Na_2_EDTA, 1.0 mM EGTA, 1.0 mM dithiothreitol, 0.1 mM phenylmethylsulfonyl fluoride and 250 mM sucrose, supplemented with protease inhibitors (10 μg/ml pepstatin A; 10 μg/ml leupeptin; 10 μg/ml aprotinin). After sat on ice for 15 minutes, the cells were homogenized and centrifuged at 1,000 *g *with Beckman Coulter Avanti-JE Centrifuge (USA) for 15 minutes at 4°C. Then the supernatants were centrifuged at 10,000 *g *(Beckman Coulter Avanti-JE Centrifuge, USA) for 15 minutes at 4°C, and the mitochondrial fractions (pellets) were resuspended with buffer A. The supernatants prepared at 10,000 *g *were further centrifuged at 100,000 *g *with Beckman Coulter Optima ™ L-100 XP Ultientrifuge (USA) for one hour at 4°C. The cytosol fractions (supernatants) and mitochondrial fractions were stored at -80°C. Proteins (25 μg) extracted from the cytosol and mitochondria were separated by 15% SDS-polyacrylamide gel electrophoresis and transferred onto nitrocellulose membranes. Membranes were blocked with 5% nonfat dry milk in Tris-buffered saline containing 0.01% Tween 20 and incubated with mouse anti-cytochrome c monoclonal antibody (BD bioscience, USA). β-actin (1:2000, Sigma, USA) was used as internal control. Blots were washed, incubated with goat anti-mouse IgG conjugated to horseradish peroxidase and developed by incubation with enhanced chemiluminescence Western blot detection reagents (Amersham, USA).

For the detection of caspase-3, the cells were lysed with buffer A. The lysed cells were centrifuged at 12 000 *g *with Beckman Coulter Avanti-JE Centrifuge (USA). Equal amounts of protein (40 μg) were used and Western blot analysis was performed following the same procedure as the detection of cytochorome c. The monoclonal antibody against caspase-3 (Calbiochem, USA) was used. β-actin (1:2000, Sigma, USA) was used as internal control.

### Detection of superoxide production

Cardiomyocytes (2 × 10^5 ^cells) were plated on 24-well plates with a 12 mm glass coverslip precoated with poly-L-lysine (10 μg/mL). Hydroethidine (HEt, Polyscience), a reduced derivative of ethidium bromide, can cross cell membrane and be oxidized by superoxide (O_2_^-^) specifically, yielding red fluorescent ethidium bromide tightly binding to DNA. The intracellular superoxide production was measured from the fluorescence of HEt. After the hypoxia-reoxygenation experiments, the cells were incubated with 1 g/ml HEt for 15 minutes in dark at 37°C and then incubated for one hour in 4% (wt/vol) paraformaldehyde in PBS for fixation and washed with PBS. The cover slips were mounted onto glass slides and these slides were observed with a fluorescent microscope (Axioskop 2-plus, Zeiss, USA) at excitation wavelength between 510 nm and 550 nm and an emission wavelength at >580 nm.

### Statistical analysis

All data were expressed as mean ± standard deviation (SD). One way ANOVA was used to assess the statistical significance of differences and followed by Student-Newman-Keuls (S-N-K) test for two group comparisons. SPSS 16.0 (IBM, USA) was used to perform all stastical analysis. *P *< 0.05 was considered as statistically significant.

## Results

### Effects of EGb761, quercetin and bilobalide on apoptotic cell death

**Figure 1:**
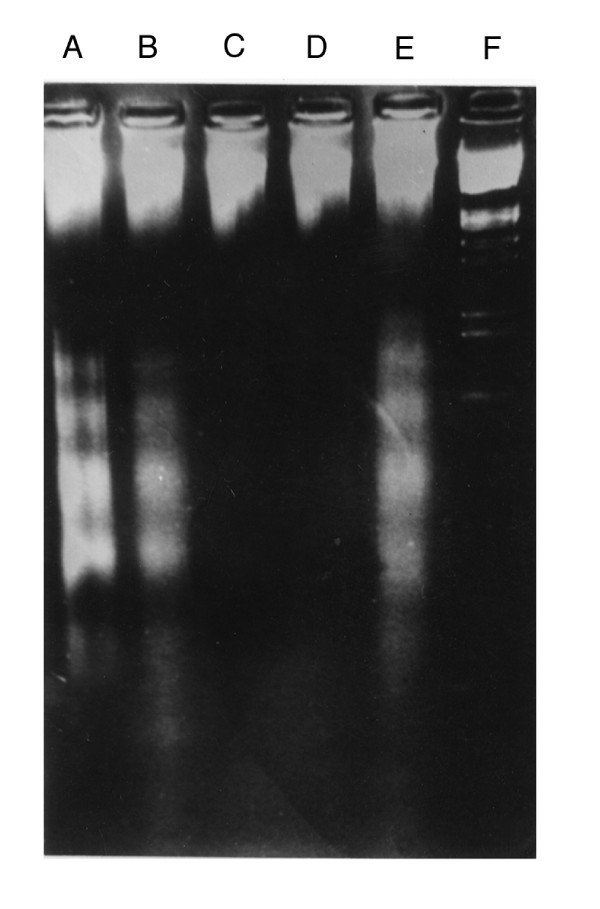
**Effects of EGb761, quecertin and bilobalide on DNA fragmentations in the hypoxia-reoxygenated cardiomyocytes**. DNA fragmentation was detected with agarose gel electrophoresis. (A) hypoxia-reoxygenation (HR); (B) HR + bilobalide (100 μg/ml); (C) HR + quercetin (100 μg/ml); (D) HR + EGb761 (100 μg/ml); (E) hypoxia (HO); (F) λ DNA/EcoRI+Hind III marker

**Figure 2:**
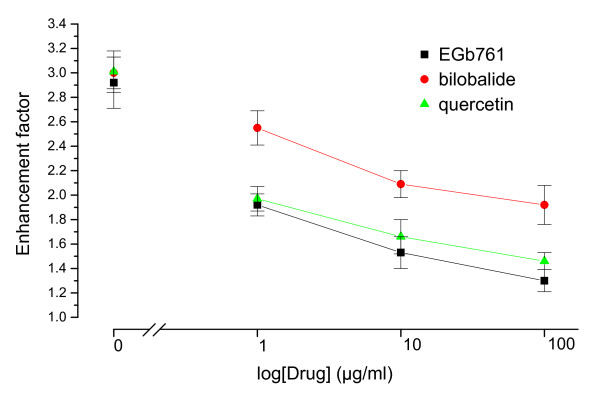
**Effects of EGb761, quecertin and bilobalide on the enrichment factors (EF) of DNA fragmentations in the hypoxia-reoxygenated cardiomyocytes**. DNA fragmentation was determined with the cell death detection ELISA method. EF represented the enrichment of histone-associated mono- and oligonucleosomes released into the cytoplasm. Drug concentrations of EGb761, quecertin and bilobalide were 1, 10, 100 μg/ml respectively. Data are shown as mean ± SD (*n *= 5).

We first investigated the inhibitory effects of EGb761, quercetin and bilobalide on apoptotic cell death in the hypoxia-reoxygenated cardiomyocytes using agarose electrophoretic analysis and cell death ELISA detection. As shown in Figure 1, the characteristic ladder pattern of the DNA fragmentation was found in the hypoxic cardiomyocytes and the DNA ladder became prodominant in the hypoxia-reoxygenated cardiomyocytes. Both EGb761 and quercetin showed inhibitory effects on the formation of the DNA fragmentation whereas bilobalide had no effect on the DNA fragmentation. We next quantitatively determined the apoptotic cell death with a cell death ELISA kit. The enrichment factor (EF) represents the enrichment of histone-associated mono- and oligo-nucleosomes released into the cytoplasm. The hypoxia treatment led to produce DNA fragmentation and the following reoxygenation treatment significantly increased the magnitude of DNA fragmentation (hypoxia: EF 1.75 ± 0.17; hypoxia-reoxygenation: EF 2.92 ± 0.21). As shown in Figure 2, EGb761, quercetin and bilobalide had inhibitory effects on the enrichment of DNA fragmentations in a dose-dependent manner. The inhibitory effects of EGb761 and quercetin were more pronounced than those of bilobalide. These results indicate that quercetin has better cardioprotective effects than bilobalide in the cardiomyocytes exposed to hypoxia-reoxygenation condition.

### Effects of EGb761, quercetin and bilobalide on the release of cytochrome c

**Figure 3:**
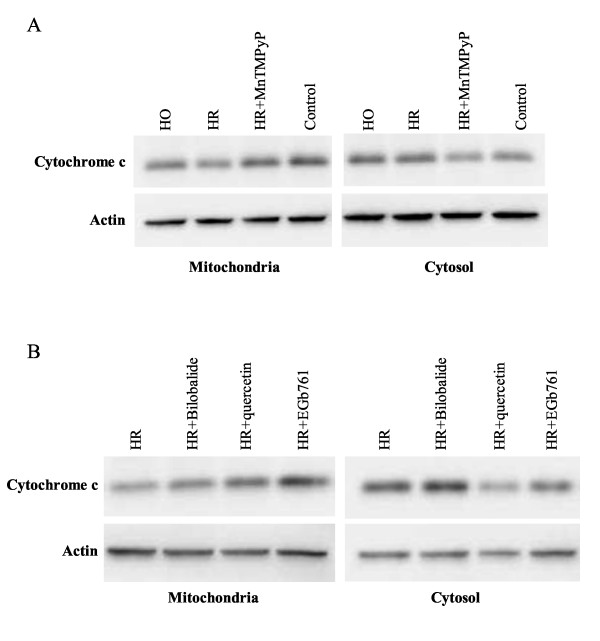
**Effects of EGb761, quecertin and bilobalide on the release of cytochrome c from mitochondria in the hypoxia-reoxygenated cardiomyocytes**. Cytochrome c was detected by Western blot analysis using a monoclonal antibody against cytochrome c. Anti-actin antibody was used as internal control. (A) effects of MnTMPyP (5 μM) on the release of cytochrome c in hypoxia-reoxygenated cardiomyocytes; (B) effects of EGb761, quecertin and bilobalide on the release of cytochrome c in hypoxia-reoxygenated cardiomyocytes. HO: Hypoxia; HR: hypoxia-reoxygenation. Drug concentration of EGb761, quecertin and bilobalide was 100 μg/ml. These representative data were obtained from three independent experiments.

We then determined whether EGb761, quercetin and bilobalide could reduce the release of cytochrome c from mitochondria, an important apoptotic pathway triggered by caspases in the hypoxia-reoxygenated cardiomyocytes. Figure 3 illustrates the results of immunoblot analysis on the expression of cytosol and mitochondrial cytochrome c. The expression of cytochrome c was predominantly distributed in the mitochondrial fractions instead of the cytosol fractions in the cardiomyocytes under normoxia condition; however, after the cardiomyocytes were exposed to hypoxia for 24 hours, the expression of cytochrome c in the cytosol fractions became predominant, indicating that the hypoxic treatment induces the release of cytochrome c from the mitochondria. Compared with the hypoxia group, the cardiomyocytes treated with twenty-four hours of hypoxia following four hours of reoxygenation had a significantly enhanced release of cytochrome c from the mitochondria. Importantly, the treatment of EGb761 markedly inhibited the release of cytochrome c from the mitochondria. We also compared the effects of EGb761 and its ingredients quercetin and bilobalide on the release of cytochrome c after the hypoxia-reoxygenation treatment. The results showed that EGb761 and quercetin had much better effects than bilobalide. In addition, as a positive control, MnTMPyP, a SOD mimic to dismutate cellular superoxide, was added to culture medium. Pre-treatment of MnTMPyP also inhibited the release of cytochrome c from the cardiomyocytes after the hypoxia-reoxygenation treatment. These results suggest that the inhibitory effects of EGb761 on the release of cytochrome c are mainly attributed to the antioxidant constitutions or their synergetic actions with terpenoid components in EGb761.

### Effects of EGb761, quercetin and bilobalides on caspases activities

**Figure 4:**
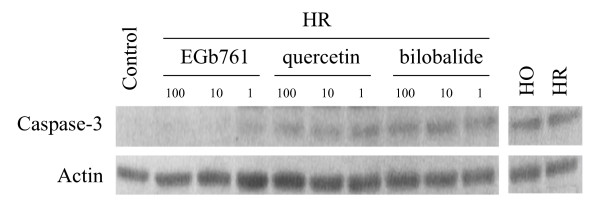
**Effects of EGb761, quecertin and bilobalide on the expression of caspase-3 in the hypoxia-reoxygenated cardiomyocytes**. Caspase-3 was detected with Western blot analysis using the monoclonal antibody against caspase-3. Anti-actin antibody was used as internal control. HO: hypoxia; HR: hypoxia-reoxygenation. Drug concentrations of EGb761, quecertin and bilobalide were 1, 10 and 100 μg/ml respectively. These representative data were obtained from five independent experiments.

**Figure 5:**
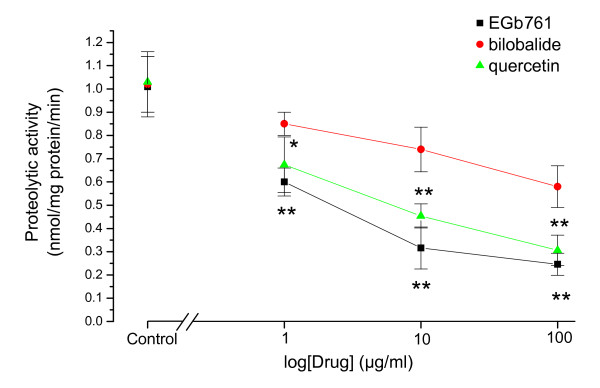
**Effects of EGb761, quecertin and bilobalide on the cleavage activities of caspases in the hypoxia-reoxygenated cardiomyocytes**. The extracted proteins from the cardiomyocytes were incubated with Ac-DEVD-AMC and the release of 7-amino-4-methylcoumarin (AMC) was measured. Drug concentrations of EGb761, quecertin and bilobalide were 1, 10 and 100 μg/ml respectively. HO: hypoxia; HR: hypoxia-reoxygenation. Data are shown as mean ± SD (*n *= 5).

Next we investigated the cleavage activities of caspases and the expression of caspase-3 in the hypoxia-reoxyegenated cardiomyocytes. Ac-DEVD-AMC is a fluorogenic tetrapeptide substrate cleaved by caspases. The results showed that the Ac-DEVD-AMC cleaving activity was significantly elevated in the groups of both hypoxia treatment and hypoxia-reoxygenation treatment (control: 0.12 ± 0.04; hypoxia: 0.78 ± 0.06; hypoxia-reoxygenation: 1.02 ± 0.12 nmol/mg protein/min). Figure 4 shows the effects of EGb761, quercetin and bilobalide on the cleavage activities of caspases. EGb761, quercetin and bilobalides decreased the Ac-DEVD-AMC cleaving activities in the hypoxia-reoxygenated cardiomyocytes in a dose-dependent manner. Both EGb761 and quercetin had stronger inhibitory effects on the Ac-DEVD-AMC cleaving activities than bilobalide. Moreover, the expression of caspase-3 was detected with Western blot analysis using anti-caspase-3 antibody (Figure 5). The expression of caspase-3 was clearly enhanced in both hypoxic and hypoxia-reoxygenated cardiomyocytes. EGb761, quercetin and bilobalide had dose-dependent inhibitory effects on the expression of caspase-3 in the hypoxia-reoxygenated cardiomyocytes. Importantly, EGb761 had stronger inhibitory effects than either quercetin or bilobalide alone, indicating that the synergetic action of flavonoids and terpenoids may contribute to inhibitions of EGb761 on the activations of caspase pathway in the hypoxia-reoxygenated cardiomyocytes.

### Effects of EGb761, quercetin and bilobalides on scavenging superoxide anions

**Figure 6:**
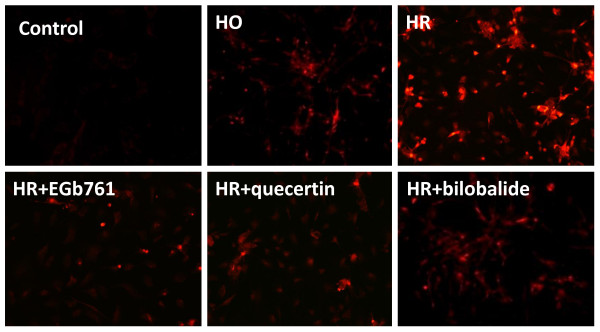
**Effects of EGb761, quecertin and bilobalide on the production of O**_**2**_^- ^**in the hypoxia-reoxygenated cardiomyocytes**. The production of O_2_^- ^in the cardiomyocytes was visualized using HEt-staining fluorescent imaging technology. HO: hypoxia; HR: hypoxia-reoxygenation. The concentration of EGb761, quecertin and bilobalide was 100 μg/ml. These representative data were obtained from three independent experiments.

We finally visualized the production of O_2_^- ^in the cardiomyocytes using HEt-staining fluorescent imaging technology. As shown in Figure 6, hypoxia-reoxygenation treatment markedly induced the HEt fluorescence, indicating the production of O_2_^-^. Both EGb761 and quercetin significantly reduces the production of O_2_^- ^in the hypoxia-reoxygenated cardiomyocytes; however, bilobalides had no significant effects on the production of O_2_^-^. Taken together, these results suggest that the antioxidant properties of EGb761 contribute to the anti-apoptotic effects on the hypoxia-reoxygenated cardiomyocytes. The anti-apoptotic effects of EGb761 are partially attributed to scavenge superoxide and inhibit the release of cytochrome c from the mitochondria in the hypoxia-reoxygenated cardiomyocytes.

## Discussion

In the experiments of this study, hypoxia-reoxygenation induced the production of superoxide, the release of cytochrome c from mitochrondria, up-regulation of caspase-3, activated the cleavage activities of caspases and triggered apoptotic cell death in the cardiomyocytes. EGb761 exhibited cardioprotective effects on scavenging superoxide, inhibited the release of cytochrome c from the mitochrondria and the activation of the cleavage activities of caspases, and prevented apoptotic cell death in the hypoxia-reoxygenated cardiomyocytes. EGb761 and quercetin had stronger inhibitory effects than the terpenoid component bilobalide. MnTMPyP, a SOD mimic to dismutate cellular superoxide, also inhibited the release of cytochrome c from mitochondria in the cardiomyocytes after the hypoxia-reoxygenation treatment. Therefore, we hypothesize that the antioxidant components of EGb761 contribute to the cardioprotective effects of EGb761 *via *the regulation of the mitochondria-dependent caspases pathways. To our knowledge, the present study provides the first evidence that EGb761 and its antioxidant components have inhibitory effects on the release of cytochrome c from the mitochondria, activation of caspases and apoptotic cell death in cardiomyocytes under hypoxia-reoxygenation conditions.

Exposure to hypoxia for 24 hours induced the occurrences of DNA fragmentation, a characteristic of apoptotic cell death. Treatment of hypoxia for 24 hours followed by four hours of reoxygenation further increased the magnitude of DNA fragmentation, suggesting reoxygenation exacerbates hypoxia-induced cell death. In the experiments, hypoxia was accompanied by the deprivation of glucose and serum whereas reoxygenation was co-supplied with glucose and serum, similar to the ischemia-reperfusion process. Our results obtained from the cultured cardiomyocytes are consistent with previous reports using various myocardial ischemia-reperfusion models [18,19].

Cytochrome c is an essential component of mitochondrial respiratory chain and released from the mitochondria in response to various stimuli. Cytosolic cytochrome c activates caspase-9 cleavages, triggering the activation of caspase 3 and apoptosis [20]. To clarify the mitochondrial mechanisms of cell death in the ischemia-reperfused cardiomyocytes, we investigated the release of cytochrome c and the cleavage activation of caspases. The release of cytochrome c into cytosol was found in the cardiomyocytes exposed to hypoxia for twenty-four hours and low glucose and low serum; it became pronounced in the cardiomyocytes after four hours of reoxygenation. The expression of caspase-3 and the cleavage activation of caspases were concomitant with the release of cytochrome c, consistent with previous studies [21].

Acting as an inhibitor of several reactive oxygen species, EGb761 exhibits a wide spectrum of antioxidant activities [22]. Our previous studies and those of others suggest that the antioxidant properties of EGb761 contribute to the cardioprotective effects against myocardial ischemia-reperfusion injury [2,3,23]. In the present study, EGb761 inhibited the release of cytochrome c from mitochondria and the cleavage activities of caspases, down-regulated the expression of caspase-3 and prevented apoptotic cell death in the hypoxia-reoxygenated cardiomyocytes. EGb761 contains both antioxidant and non-antioxidant ingredients. Our data clearly showed that quercetin, an antioxant in EGb761, had stronger inhibitory effects than bilobalide on the release of cytochrome c, the activation of caspases and apoptotic cell death in the hypoxia-reoxygenated cardiomyocytes. Quercetin showed a similar inhibitory effect as EGb761, suggesting that ginkgo-flavone glycosides may be the major components of EGb761, contributing to its cardioprotective effects on inhibiting the release of cytochrome c and activation of caspases. These results are consistent with the report that flavonoid components exhibit better effects than Ginkgolides and Bilobalides on protections of mitochondrial membrane potential in NO-treated PC12 cells and mouse brain cells [24].

It is necessary to mention that bilobalide also inhibited the mitochrondria-dependent caspases activation and prevented the cardiomyocytes from apoptotic cell death even though the effects of bilobalide were much weaker than those of quercetin. A previous report suggested that ginkgolides, the non-flavon fraction of EGb761, protected PC12 cells against hypoxia-induced injury *via *p22/p44 MAPK pathway dependent elevation of HIF-1 transcription efficiency [25]. Therefore, the synergetic actions of flavone glycosides and terpenoids may contribute to the cardioprotective effects of EGb761 on inhibiting the release of cytochrome c and activation of caspases and preventing cell death during myocardial ischemia-reperfusion injury.

## Conclusion

The antioxidant constituents such as quercetin mainly contribute to the cardioprotective effects of EGb761 and inhibit the mitochondria-dependent caspase pathway. It is possible that the mitochondria-dependent caspase pathway may be one of the molecular targets of EGb761 against myocardial ischemia-reperfusion injury.

## Abbreviations

HO: hypoxia; HR: hypoxia-reoxygenation; ROS: Reactive oxygen species; NO: nitric oxide; Apaf: apoptosis protease activating factor; DMEM: Dulbecco's modified Eagle's medium; PBS: phosphate buffered saline; FCS: fetal calf serum; Het: hydroethidine; EF: enrichment factor; SOD: Superoxide dismutase; AMC: 7-amino-4-methylcoumarin; EGTA: sodium ethylene glycol tetraacetic; Na_2_EDTA: sodium ethylenediaminetetracetic; SD: standard deviation

## Competing interests

The authors declare that they have no competing interests.

## Authors' contributions

JS designed the study, performed the experiments, analyzed the data and drafted the manuscript. WL performed the experiments and analyzed the data. YG collected the background materials and drafted the manuscript. YT, PCWF and LT interpreted the results and revised the manuscript. All authors read and approved the final version of the manuscript.
